# Water Uptake Tradeoffs of Dominant Shrub Species in the Coastal Wetlands of the Yellow River Delta, China

**DOI:** 10.3389/fpls.2022.935025

**Published:** 2022-06-23

**Authors:** Jinfang Zhu, Jingtao Liu, Junsheng Li, Caiyun Zhao, Jingkuan Sun

**Affiliations:** ^1^State Key Laboratory of Environmental Criteria and Risk Assessment, Chinese Research Academy of Environmental Sciences, Beijing, China; ^2^Shandong Provincial Key Laboratory of Eco-Environmental Science for Yellow River Delta, Binzhou University, Binzhou, China

**Keywords:** water use strategy, available soil water, water source, stable oxygen isotope, wetland plants

## Abstract

*Tamarix chinensis* and *Ziziphus jujuba* are two dominant shrub species on Chenier Island in the Yellow River Delta, China. Water is a restrictive factor determining the plant growth, vegetation composition, and community succession in this coastal zone. We investigated how water uptake tradeoffs of the two shrub species responded to soil water fluctuations caused by seasonal variations of precipitation. The soil water content, salinity and δ^18^O values of potential water sources (soil water in 0–20, 20–40, 40–60, and 60–100 cm soil layers, and groundwater) and plant xylem water were measured in wet (July 2013) and dry (July 2014) seasons. The IsoSource model was employed to calculate the contributions of different water sources to plant xylem water. The results showed that δ^18^O values of soil water decreased significantly with soil depth in the dry season, while increased significantly with soil depth in the wet season. In the wet season, when the soil water was abundant, *Z. jujuba* mostly used the soil water from the 60–100 cm layer, while *T. chinensis* took up a mixture of groundwater and soil water from the 60–100 cm layer. In the dry season, when the soil water was depleted because of low precipitation, *Z. jujuba* mainly took up a mixture of the soil water from 20 to 100 cm soil layers, while *T. chinensis* mainly used groundwater. *T. chinensis* and *Z. jujuba* showed different ecological amplitudes of water sources during dry and wet seasons. The niche differentiation of major water sources for *T. chinensis* and *Z. jujuba* demonstrated their adaptabilities to the fluctuations of soil moisture in water-limited ecosystems. Water niche differentiations of coexisting shrub species were expected to minimize their competition for limited water sources, contributing to successful coexistence and increasing the resilience of the coastal wetland ecosystem to drought.

## Introduction

Available water is a key determinant of vegetation composition and community succession (Gazis and Feng, 2004; Xu et al., 2011; Wei et al., 2013; Xia et al., 2014). In coastal wetlands, the available water is extremely limited due to the increasing salinity of soil water and groundwater induced by the seawater intrusion, and this restrict the growth of non-halophytic plants significantly (Marcelis and Van Hooijdonk, 1999; Osland et al., 2011; Chandrajith et al., 2013; Janousek and Mayo, 2013; Lu et al., 2020). Uneven and stochastic precipitation usually causes temporal–spatial heterogeneity in water availability, increasing the scarcity of the already limited availability (Drake and Franks, 2003; Wu et al., 2014; Gu et al., 2015). Because of the limitation of available water, competition among species in coastal wetlands is likely to be more intense (Asbjornsen et al., 2007; Ewe et al., 2007). This competition is weakened when plants use different water sources (e.g., soil water, groundwater, rainwater, and seawater) (Sternberg and Swart, 1987; Sternberg et al., 1991; Ewe et al., 1999; Corbin et al., 2005). In addition, plant species can adapt to water stress by shifting their main water sources as the soil water conditions change (Ewe et al., 1999; Zhu et al., 2018). Therefore, the tradeoffs in plant water uptake play a critical role in the successful coexistence of dominant species with similar life forms.

Plant water uptake is closely related to the distribution patterns of root systems (Dawson and Pate, 1996; Oliveira et al., 2005; Aroca et al., 2012; Wang et al., 2015). Shallow-rooted plants mainly absorb water from surface soil, and deep-rooted plants can exploit deeper soil water and even groundwater (Williams and Ehleringer, 2000; Yang et al., 2011; Rossatto et al., 2012; Wu et al., 2016). However, some previous studies have shown that the proportions of soil water from different depths for a plant species are not exactly consistent with the distribution patterns of that plant’s root systems (Dawson and Pate, 1996; Chimner and Cooper, 2004). This phenomenon is caused by the variations in available water along the soil profile, as the plant often first relies on more dependable water sources. Dawson and Pate’s (1996) results showed that plants derived the majority of the water they use from deeper sources via their taproots in dry season, while in the wet season most of the water they use was derived from shallower soil water via their lateral roots. The ability to exploit deeper, dependable water sources makes it possible for plants to survive a long dry period without precipitation (Ehleringer et al., 1991; Dawson and Pate, 1996; Wu et al., 2014; Dai et al., 2015). In coastal wetlands, salinity is also an important factor determining the water uptake patterns of plants (Ewe et al., 2007). A previous study reported that halophytic species were able to use deeper soil water, or groundwater with high salinity, but non-halophytic species depended only on soil water with lower salinity or rainwater stored in the soil (Zhu et al., 2018). The difference in the water sources used by plants contributes to water niche differentiation, which is the key to their successful coexistence.

Chenier Island, one of the world’s three large, ancient shell ridges, formed in the Yellow River Delta (YRD) by the accumulation of shells over thousands of years (Zhao et al., 2015). The coastal wetland ecosystem on Chenier Island plays a key role in protection against coastal erosion. It also provides habitats as resting grounds for some migratory bird species and as breeding grounds for others (Zhao et al., 2015). Precipitation is the main freshwater source of soil water recharge and determines the vegetation composition and distribution patterns in this region. Against the background of global climate change, however, precipitation is becoming more uneven and shows significant annual differences which may cause frequent drought (Bai et al., 2020). The movement of precipitation through the soil profile is significantly affected by soil texture, characterized by coarse particle diameter, large porosity, and low water conservation in this area (Zhu et al., 2018). Because of the impact of large porosity, most of the precipitation infiltrates into deep soil layers or groundwater immediately after it falls, and only a small proportion is short-lived in the soil profile available for plant water use (Zhu et al., 2016). On Chenier Island, therefore, soil water is the critical factor limiting the growth and distribution of plants, and its availability will become an even more significant factor in dry seasons with little precipitation. The allocation of water sources is very important to plant survival through a long dry period in this region. Understanding the water tradeoff of dominant plant species is beneficial to the protection and restoration of the coastal wetland ecosystem.

*Tamarix chinensis* and *Ziziphus jujuba* are two typical xerophytic shrub species that are mainly distributed in the arid and semi-arid areas of China. They have similar life forms and high tolerance to drought stress (Zhu et al., 2018). *T. chinensis*, a common shrub species in YRD, has higher tolerance for salt stress (Zhu et al., 2015). On Chenier Island, *Z. jujuba* and *T. chinensis* are two dominant shrub species coexisting on the dune crest (Zhu et al., 2018). They are ecologically important in protecting the coast from wind and waves (Zhao et al., 2015). The result of our previous study (Zhu et al., 2018) showed that the two species had different water use patterns during growing seasons. Little was known, however, about their water use strategies in wet and dry seasons. Hence, a field experiment was carried out in the same month in different years to investigate (1) whether the two shrub species have differences in water use strategies, and (2) how they shift water sources to adapt to the fluctuations in soil water resulting from precipitation. We hypothesized that (1) *T. chinensis* and *Z. jujuba* have different water use patterns in dry and wet seasons; (2) *T. chinensis*, which has higher salt tolerance than *Z. jujuba*, may use deeper water sources than *Z. jujuba* when the soil water is limited in the dry season.

## Materials and Methods

### Study Area

**FIGURE 1:**
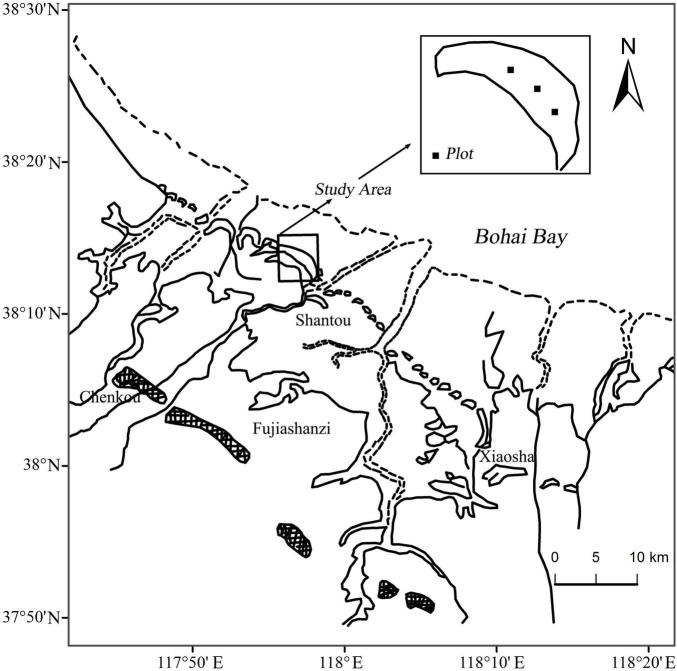
Location map of the study area and sampling sites.

The study was conducted on Chenier Island lies on the Binzhou National Shell Ridge and Wetland Nature Reserve in the YRD (38°05′–38°21′N, 117°46′–118°05′E) along the northern coast of Wudi county, Shandong province, China (Figure 1). A typical temperate continental monsoon climate dominates in the study area. Mean annual temperature is 12.4°C. Mean annual precipitation and mean annual evaporation are 552.4 and 2430.6 mm, respectively (Zhao et al., 2015; Zhu et al., 2016, 2018). Seventy percent of annual precipitation happens between June and September (Zhu et al., 2018). The study area is formed by long-term accumulation of shells brought by tides. The soil matrix consists of shells, mud and sand, and the soil texture is characterized by large porosity, weak water, and fertilizer holding capacity (Zhao et al., 2015). The soil types are mainly shell sand soil and coastal saline soil, and coastal saline soil is dominant on the seaward and landward sides (Zhu et al., 2015; Chen et al., 2019). The groundwater level is very shallow, only 1.8 m depth.

*T. chinensis* and *Z. jujuba* are two dominant shrub species in the local vegetation communities with the coverage of 75∼90%. The root architectures are clearly different between two shrub species. The root length of *Z. jujuba* is about 1.5 m, with mainly shallow root system. The root length of *T. chinensis* is about 2.2 m, with mainly vertical root system. Associated plants are mainly herbaceous species with shallow roots, such as *Artemisia mongolica* Fisch., *Astragalus adsurgens* Pall., *Messerschmidia sibirica* Linn., *Cayratia japonica* (Thunb.) Gagnep., *Apocynum venetum* L., and *Heteropappus altaicus* (Willd.) Novopokr.

### Sample Collection

We conducted a field experiment in shrubland dominated by *T. chinensis* and *Z. jujuba* on Chenier Island in the YRD, China, in July 2013 (wet season) and July 2014 (dry season). Three 10 m × 10 m sample plots were established as the permanent plots in the study area. All plant, soil and groundwater samples were collected on clear days over 5 days after the last rainfall at the end of July 2013 and July 2014. Fully suberized twigs were randomly selected and cut from each species for of xylem water extraction. There were three replicates for each species in each plot. Soil samples were collected for stable isotope, soil water content (SWC) and salinity analyses. Three soil profiles were chosen randomly from different directions within 1 m of the sampled plants in each plot. The soil samples were collected at depths of 0–20, 20–40, 40–60, and 60–100 cm by using a soil auger, respectively. Three groundwater samples were pumped using a 200 cm vitrified-clay tube fixed within 1 m of the sampled plants in each plot. All samples were collected between 06:00 and 08:00 a.m.

To avoid isotopic fractionation caused by evaporation, all samples were placed in individual screw-capped glass vials immediately after collection, sealed with Parafilm and stored in a freezer before water extraction. The soil samples that were used to analyze the SWC were sealed and taken back to the laboratory for determination.

The SWC was determined by the oven-drying method (Gu et al., 2015). Soil and groundwater salinity were measured by the gravimetric method. All laboratory experiments were conducted at Shandong Provincial Key Laboratory of Eco-Environmental Science for Yellow River Delta.

Climatic data of precipitation were continuously recorded at an automatic climate station on Chenier Island which was installed by Shandong Provincial Key Laboratory of Eco-Environmental Science for Yellow River Delta.

### Stable Isotope Analysis

Many studies have shown that natural stable isotopes of hydrogen and oxygen are used successfully to trace the water sources of plants (Dawson and Pate, 1996; Ehleringer et al., 2000; Ewe et al., 2007; Wu et al., 2014; Gu et al., 2015), as different water sources have different isotopic compositions because of the isotopic fractionation of hydrogen and oxygen during physical processes (e.g., evaporation and condense) (Ehleringer and Dawson, 1992). The application of isotopic technology on plant water use is based on the assumption that there is no isotopic fractionation of hydrogen and oxygen during water uptake by plant roots (Ewe et al., 2007; Meißner et al., 2014). In most ecosystems the isotopic composition of plant xylem water can reflect the isotopic signatures of water around the root system. However, some studies have shown that certain halophytic or xerophytic plants can fractionate hydrogen isotopes significantly during water uptake, while oxygen isotopic fractionation is negligible during root water uptake (Sternberg and Swart, 1987; Lin and Sternberg, 1993; Ellsworth and Williams, 2007). Consequently, in this study, we used oxygen isotopes to determine plant water uptake patterns. Plant xylem water and soil water were extracted using a cryogenic vacuum distillation system, and the extracted water samples were stored in screw-cap glass vials at 4°C for isotope determination (Zhu et al., 2018). The oxygen isotope ratios (δ^18^O) of the water samples were determined using a liquid water isotope analyzer (LWIA, DLT-100, Los Gates Research Inc., Mountain View, CA, United States) at the Shandong Provincial Key Laboratory of Eco-Environment Science for the Yellow River Delta. The precision of δ^18^O analysis was ±0.25‰. The oxygen isotopic composition can be expressed (Equation 1) as the O isotope ratio (δ^18^O, ‰):


(1) δO18=(R/s⁢a⁢m⁢p⁢l⁢eR-s⁢t⁢a⁢n⁢d⁢a⁢r⁢d1)×1000

where *R*_*sample*_ and *R*_*standard*_ are the ^18^O/^16^O molar ratio of the sample and the standard water (Vienna Standard Mean Ocean Water).

Extracted water of plant often contains organic materials that may cause spectral contamination when using isotope ratio infrared spectroscopy, which result in errors in the measured isotope ratios. In order to eliminate spectral contamination, a calibration curve was used to correct the δ^18^O values of the plant xylem water following the method presented by Schultz et al. (2011). The detailed procedures were described in Zhu et al. (2016).

### Data Analysis

The feasible contributions of potential water sources to plant xylem water were calculated using the IsoSource mixing model (Phillips and Gregg, 2003). In this study, the increment and the tolerance were set to 1% and 0.05‰ in our calculation, respectively. The mean and possible range of water source proportions were calculated for both species in dry and wet seasons (Phillips and Gregg, 2003; Zhu et al., 2018).

One-way analysis of variance (ANOVA) was used to detect significant differences in salinity of soil and groundwater, SWC of different soil layers or δ^18^O values of potential water sources samples in dry or wet seasons (*P* < 0.05). The differences in salinity, SWC or δ^18^O values of water sources and plant xylem water between wet and dry seasons were also compared using ANOVA (*P* < 0.05). All statistical analyses of data were performed using SPSS 17.0 (SPSS Inc., Chicago, IL, United States). The charting was processed by Origin 8.5 (Origin Lab Corp., Northampton, MA, United States).

## Results

### Precipitation Distribution

**FIGURE 2:**
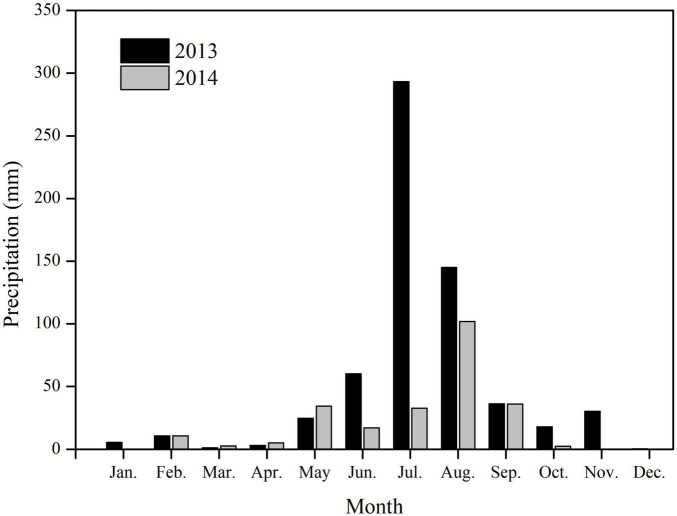
Monthly precipitations for 2013 and 2014 on Chenier Island, Yellow River Delta, China.

In the study area, the precipitation showed significant monthly differences, most occurring in summer (Figure 2). The total precipitation for 2013 was 629.0 mm, 79.2% occurring during June–August. In 2013, the maximum monthly precipitation was 293.3 mm, occurring in July. In 2014, the total precipitation was 243.6 mm, 91.1% occurring during May–September. Precipitation in July 2014 was only 32.6 mm, significantly lower than that in July 2013 (Figure 2).

### Soil Water Content

**FIGURE 3:**
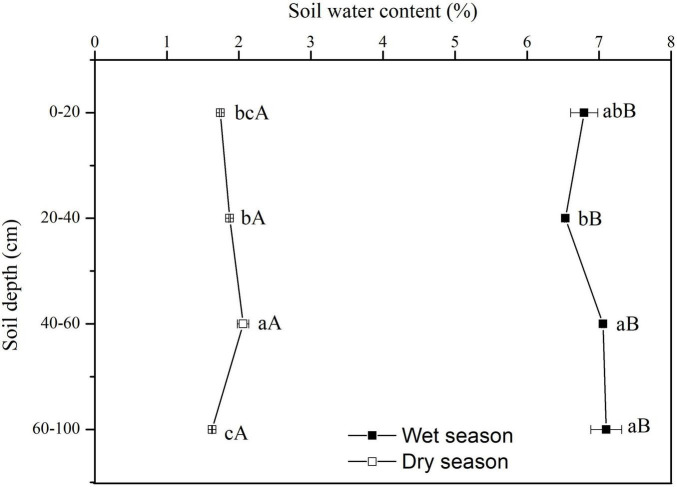
Variation in vertical profile of soil water content (SWC) in dry and wet seasons. Error bars represent standard errors of mean SWC (*n* = 9). Different lowercase letters represent the significant differences in SWC among different soil depths in dry or wet seasons at the 0.05 level. Different capital letters represent significant differences in SWC at each soil depth between dry and wet seasons at the 0.05 level.

The SWC showed significant vertical variations in the dry season (*F* = 21.08, *P* < 0.01). There was no significant vertical variation in SWC along the soil profile in the wet season (*F* = 3.36, *P* = 0.076) (Figure 3). In the dry season, the SWC rose significantly with increasing depth up to 60 cm, but at depths of 60–100 cm the SWC was only 1.627%, and it was significantly lower than that in upper soil layers (*P* < 0.05). In the wet season, the SWC increased gradually along the soil profile. All SWC values at each soil depth in the wet season were significantly higher than at the same soil depth in the dry season (*P* < 0.05) (Figure 3).

### Salinity of Soil and Groundwater

Soil salinity showed significant vertical variations in dry (*F* = 10.69, *P* < 0.01) and wet (*F* = 9.72, *P* < 0.01) seasons (Table 1). In the dry season, the salinity of soil water increased first and then decreased, and there were no significant differences in soil salinity at depths of 0–60 cm (*P* > 0.05). The soil salinity at 60–100 cm was significantly lower than at 0–60 cm depth(*P* < 0.05). In the wet season, the soil salinity increased significantly along the soil profile, and the soil salinity of each soil layer and groundwater were higher than in the dry season (*P* < 0.05). The salinity of groundwater in both seasons was significantly higher than that of the soil (*P* < 0.05) (Table 1).

**TABLE 1:** Salinity of soil and groundwater in dry and wet seasons (average ± standard error).

Water sources		Salinity (%)	
		Wet season	Dry season
Soil depth (cm)	0–20	0.0850 ± 0.0014cA	0.0672 ± 0.0015aB
	20–40	0.0925 ± 0.0014bcA	0.0724 ± 0.0028aB
	40–60	0.0942 ± 0.0022bA	0.0665 ± 0.0021aB
	60–100	0.1058 ± 0.0046aA	0.0573 ± 0.0001bB
Groundwater		1.7454 ± 0.0096A	1.9400 ± 0.0058B

*Different lowercase letters represent significant differences in soil salinity at different soil depths in dry or wet season at the 0.05 level. Different capital letters represent significant differences in soil salinity at each soil depth and groundwater between dry and wet seasons at the 0.05 level.*

### δ^18^O Values of Soil Water, Groundwater, and Xylem Water

**FIGURE 4:**
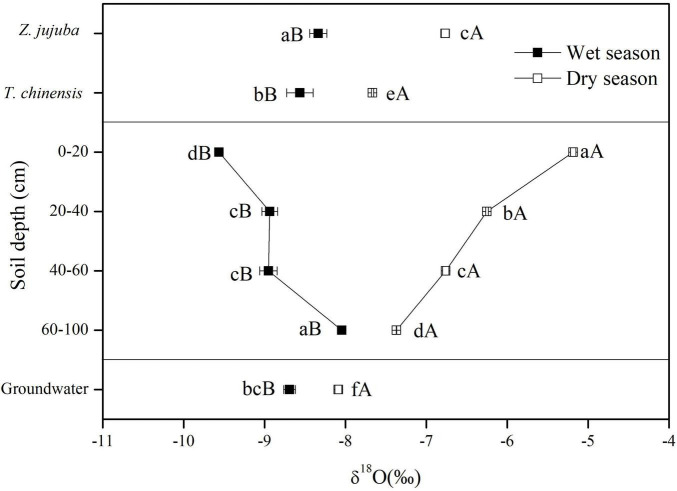
δ^18^O values of potential water sources and plant xylem water for dry and wet seasons. Error bars represent standard errors of mean δ^18^O (*n* = 9). Different lowercase letters represent the significant difference in δ^18^O values among different samples in dry or wet seasons at the 0.05 level. Different capital letters represent significant difference in δ^18^O values of each sample between dry and wet seasons at the 0.05 level.

The δ^18^O values of soil water showed clear vertical and seasonal variations (Figure 4). In the wet season, the δ^18^O values of soil water increased significantly with increasing soil depth, and reached a maximum at 60–100 cm. However, there was no significant difference in δ^18^O values between soil layers of 20–40 cm and 40–60 cm, with an average of −8.95‰ (*P* > 0.05). In the dry season, the δ^18^O values of soil water showed an opposite vertical variation: decreasing with increasing soil depth. There were significant differences in the δ^18^O values of soil water among all soil layers (*P* < 0.05). The δ^18^O values of soil water in the wet season were significantly lower than those in the dry season (*P* < 0.05), and the differences in δ^18^O values of soil water at the same soil layer between wet and dry seasons decreased with increasing soil depth.

The δ^18^O values of groundwater in the wet season were more negative than those in the dry season. In the wet season the δ^18^O values of groundwater were significantly lower than those of soil water at 60–100 cm (*P* < 0.05), but they were higher than those of soil water at other soil layers. In dry seasons the δ^18^O values of groundwater were significantly lower than those of soil water at all soil layers (*P* < 0.05) (Figure 4).

The δ^18^O values of xylem water also showed seasonal variations (Figure 4). The δ^18^O values of xylem water for both species in the dry season were significantly higher than those in the wet season (*P* < 0.05). In the wet season the δ^18^O values of xylem water for *Z. jujuba* were close to those of soil water at 60–100 cm and were not significantly different from them (*P* > 0.05). For *T. chinensis*, the δ^18^O values of xylem water were intermediate between those of soil water at 60–100 cm and groundwater, and were not significantly different from those of groundwater in the wet season (*P* > 0.05). In the dry season, the δ^18^O values of xylem water for *Z. jujuba* were close to those of soil water at 40–60 cm, and those of xylem water for *T. chinensis* were intermediate between those of soil water at 60–100 cm and groundwater. The δ^18^O values of xylem water for *Z. jujuba* were all more enriched than those of xylem water for *T. chinensis* in both seasons (Figure 4).

### Feasible Contributions of Potential Water Sources

The proportions of potential water sources for *T. chinensis* and *Z. jujuba* showed clear seasonal variations (Table 2). *Z. jujuba* took up 82.2% of its required water from the 60–100 cm soil layer in the wet season, and only 17.8% of its xylem water was absorbed from other soil layers and groundwater. In the dry season, the proportion of soil water at 60–100 cm for *Z. jujuba* decreased, and contributions of soil water to *Z. jujuba* at other soil layers increased; about 68.3% of xylem water was absorbed from the 20–100 cm soil layer (Table 2).

**TABLE 2:** Proportions of potential water sources (%) for *Tamarix chinensis and Ziziphus jujuba* in dry and wet seasons.

Water sources		Wet season		Dry season	
		*Z. jujuba*	*T. chinensis*	*Z. jujuba*	*T. chinensis*
Soil depth (cm)	0–20	2.1 (0–11)	6.2 (0–28)	15.0 (0–47)	3.4 (0–16)
	20–40	4.4 (0–21)	12.0 (0–54)	21.8 (0–74)	5.8 (0–25)
	40–60	4.3 (0–21)	11.8 (0–52)	25.1 (0–100)	8.2 (0–35)
	60–100	82.2 (67–95)	51.2 (17–78)	21.4 (0–74)	15.6 (0–65)
Groundwater		7.0 (0-33)	18.8 (0–83)	16.8 (0–56)	67.0 (35–87)

*Mean source proportions and range of minimum and maximum source proportions (in parentheses) were calculated using the IsoSource model (Phillips and Gregg, 2003).*

For *T. chinensis*, 51.2% of its xylem water was taken up from the 60–100 cm soil layer, and 18.8% was absorbed from groundwater in the wet season (Table 2). In the dry season, *T. chinensis* usage of soil water at 60–100 cm clearly decreased and it shifted its main water sources from deep soil water to groundwater. The proportion of groundwater for *T. chinensis* was up to 67.0% (Table 2).

## Discussion

### Spatial and Temporal Variations of the Soil Water Content and Salinity

Moisture and salinity were two limiting factors for plants growth and distribution in coastal wetlands (Ewe et al., 2007; Yu et al., 2019). The transport of salt was often accompanied by the movement of water. Generally, the recharge of soil water mainly depends on two water sources: precipitation and groundwater. Precipitation is the main water input maintaining plant growth on the surface of the earth (Wu et al., 2014; Walter, 2018). The recharge level of soil water is attributed to the amount, frequency and intensity of precipitation (Walter, 2018). Previous studies have shown that heavy precipitation can recharge deep soil water, but light precipitation just has an impact on the SWC of topsoil (Xu et al., 2012; Zhu et al., 2016). We found that the SWC at 60–100 cm was significantly lower than in the upper soil layers in the dry season, because of low precipitation, which could only recharge shallow soil water.

In coastal area, groundwater is the main source of soil water and salt. It mainly recharges soil water and salt through capillary pores, and so the soil physical properties determine the recharge ability of groundwater (Loik et al., 2004; Wallender et al., 2006; Volodina and Sokolova, 2007; Ma et al., 2011; Xia et al., 2014). However, the most remarkable characteristic of the study area on Chenier Island was its relatively low soil salinity. The soil salinity was significantly lower than that of adjacent soil (1.52%) (Xie et al., 2012). This contributes to plant species survival, especially that of the non-halophytes. The main composition of soil particles – coarse shell particles and fragments—leads to minimal water adsorption and capillarity in study area (Xie et al., 2012). It is difficult, therefore, for salt to be transported through the soil profile, even though the groundwater level is shallow. Our results showed that soil salinity was significantly lower than that of groundwater in both seasons, consistent with the above conclusion. It indicated that groundwater had little effect on the soil moisture and salinity. It also demonstrated that the recruitment of soil water mainly depended on precipitation. Therefore, during seawater intrusion, it mainly affected groundwater and has less effect on soil salinity.

We also found that soil salinity in the dry season was lower than that in the wet season (Table 1). This might have been caused by the washing effect of precipitation happened from July 2013 to July 2014. At the same time, specific soil physical properties allowed precipitation to infiltrate to groundwater directly through soil pores (Zhu et al., 2016, 2018). Hence, in our study, the salinity of groundwater in the wet season was lower than in the dry season (Table 1), as a large amount of precipitation infiltrated to groundwater and diluted its salinity.

### Spatial and Temporal Variations in Isotopic Compositions of Soil Water and Groundwater

The oxygen isotopic composition of soil water was mainly affected by evaporation and precipitation (Drake and Franks, 2003; Dai et al., 2015). Evaporation causes the enrichment of ^18^O in soil water. As the depth of the soil layer increased, the influence of evaporation on the δ^18^O values of soil water decreased (Barnes and Allison, 1988). Precipitation was the main water source for soil water recharge, so the oxygen isotopic composition of rainwater and its infiltration within the soil profile affected the vertical distribution of ^18^O in soil water. Generally, the oxygen isotopic composition of precipitation was more depleted owing to Rayleigh distillation of heavy isotopes (Clark and Fritz, 1997; Gu et al., 2015). In the wet season, 293.3 mm precipitation with low δ^18^O values recharged the soil water, and recharge reduced with increasing soil depth, so the δ^18^O values of soil water rose as soil depth increased (Figure 4). This also indicated that the depletion of soil water ^18^O by precipitation was stronger than the enrichment of soil water ^18^O by evaporation in the wet season. In contrast, because the precipitation was only 32.6 mm in the dry season, evaporation became the dominant factor enriching the δ^18^O values of soil water. Therefore, the δ^18^O values of soil water were more enriched and significantly decreased with increasing soil depth in the dry season (Figure 4). Therefore, we concluded that the seasonal differences in the δ^18^O values of soil water mainly resulted from precipitation and evaporation. In addition, we also found that there were significant differences in δ^18^O values between groundwater and deep soil water in both seasons (Figure 4). It demonstrated that groundwater had little effect on the δ^18^O values of soil water. Consequently, it was difficult for seawater to affect the oxygen isotopic composition of soil water by affecting groundwater.

Previous studies have shown that the oxygen isotopic composition of groundwater is commonly maintained at a stable level (Ewe et al., 2007). However, in this study, we found that the δ^18^O values of groundwater showed a clearly seasonal variation. The δ^18^O values of groundwater in the wet season were significantly lower than those in the dry season (Figure 4). Because the soil had a coarse particle diameter and large porosity, precipitation with depleted δ^18^O values could directly recharge the groundwater, leading to a decrease in the δ^18^O values of groundwater. At the same time, precipitation in the wet season was obviously greater than that in the dry season. Consequently, precipitation became the main factor causing the seasonal difference in oxygen isotopic composition of groundwater.

### Seasonal Patterns of Plant Water Uptake

Previous studies have shown that plant species living in the same habitat often have different water uptake patterns (Ewe et al., 1999; Wu et al., 2014, 2016). This avoids water competition during drought periods when the soil water is limited; they take up similar water sources when soil water is abundant in the environment (Gu et al., 2015). For example, Ewe et al. (2007) reported that all plants used the shallow soil water in the wet season when the soil water was abundant and less saline. In the dry season, however, *Rhizophora mangle* used a soil–groundwater mix while *Cladium jamaicense* and *Sesuvium portulacastrum* continued to use shallow soil water. In this study, although the shallow soil water was abundant in the wet season, *T. chinensis* and *Z. jujuba* did not take up water from the shallow soil layers. On the contrary, *Z. jujuba* mainly used deep soil water from the 60–100 cm soil layer, and *T. chinensis* used a mixture of deep soil water (60–100 cm) and groundwater. Both dominant shrub species used the deep soil water, and this was of benefit in minimizing water competition with associated plants with shallow root systems. It also improved the use efficiency of available soil water (Yang et al., 2011).

Plant species often explore more stable water sources through extending their roots to deeper soil layers, or even to groundwater when soil water is limited in the dry season (Ewe et al., 2007; Dai et al., 2015). In our study, when the soil water became more depleted because of low precipitation in the dry season, *T. chinensis* shifted its main water sources from deep soil water to groundwater with high salinity (1.94%), indicating that this species could extend its roots to groundwater because it had a strong tolerance to salt. At the same time, *Z. jujuba*—a low salt-tolerance shrub species—found it difficult to extend its roots to deeper layers because of the influence of salt stress. It had to maximize its use of limited water sources stored in the soil profile by expanding its absorption range of soil water from 60–100 to 20–100 cm (Table 2). From the side of isotopic composition, the oxygen isotopic composition of xylem water in a plant reflects the isotopic signatures of a mixture of water sources that the plant absorbs (Ehleringer and Dawson, 1992; Ellsworth and Williams, 2007). There was a significant difference in δ^18^O values of xylem water between *T. chinensis* and *Z. jujuba* in dry season, indicating that two species had different water sources. All of these results demonstrated that the main water sources of *T. chinensis* and *Z. jujuba* were significantly different when soil water was limited in dry season, supporting our first hypothesis. It helped to avoid competition for water between *T. chinensis* and *Z. jujuba* and promote their coexistence. By comparison, we found that *T. chinensis* could take up more stable and deeper water sources (e.g., groundwater) than *Z. jujuba* in the dry season, and this supported our second hypothesis. The result also demonstrated that *T. chinensis* had stronger drought adaptability than *Z. jujuba*. *T. chinensis* and *Z. jujuba* had the ability to shift their main water sources among different potential water sources, which indicated that they had the advantage of adaptation to a water-limited coastal ecosystem (Zhu et al., 2018).

*T. chinensis* and *Z. jujuba* showed different water uptake patterns on Chenier Island, and this also supported our first hypothesis. The water uptake patterns of plants are the result of long-term adaptation to fluctuating water conditions (Yang et al., 2011). The different water usage of *T. chinensis* and *Z. jujuba* may lead to their water niche partitioning. The niche differentiation in water uptake among coexisting shrub species is expected to minimize their competition for limited water sources and increase coastal ecosystem resilience to drought (Zhu et al., 2018).

In addition, we found that the effect of salinity on *Z. jujuba* water use was greater than that on *T. chinensis* water use. Therefore, salinity will become a critical factor determining the development of the *Z. jujuba* community as sea levels rise as a result of global warming. This leads us to infer that *Z. jujuba* will gradually disappear from the study area and *T. chinensis* will become to be the only dominant shrub species in the future.

## Conclusion

In this study, the stable oxygen isotope was used to determine the water uptake of two dominant shrub species and their responses to soil water fluctuations caused by seasonal changes in precipitation. On the Chenier Island, *T. chinensis* and *Z. jujuba* had different water uptake patterns in dry and wet seasons, and they were able to shift their water sources among different potential water sources in fluctuating water environments. In the wet season, when the soil water was abundant, *Z. jujuba* mostly used the soil water from 60 to 100 cm, while *T. chinensis* took up a mixture of groundwater and soil water from 60 to 100 cm. In the dry season, when the soil water was depleted, *Z. jujuba* mainly took up soil water from the 20 to 100 cm soil layers, while *T. chinensis* mainly used groundwater. The shifts in major water sources for *T. chinensis* and *Z. jujuba* demonstrated their adaptations to the fluctuations of soil moisture in water-limited ecosystems. The interspecific differences in water uptake clarified the mechanism of coexistence between two dominant shrub species in a coastal wetland ecosystem.

## Data Availability Statement

The original contributions presented in this study are included in the article/supplementary material, further inquiries can be directed to the corresponding authors.

## Author Contributions

JZ and JTL conceived to the idea. JZ, JTL, JS, and CZ collected the samples in the field, performed the experiments, and collected the data. JZ analyzed the data and wrote the draft manuscript. JTL and JSL revised the manuscript. All authors read and approved the final manuscript.

## Conflict of Interest

The authors declare that the research was conducted in the absence of any commercial or financial relationships that could be construed as a potential conflict of interest.

## Publisher’s Note

All claims expressed in this article are solely those of the authors and do not necessarily represent those of their affiliated organizations, or those of the publisher, the editors and the reviewers. Any product that may be evaluated in this article, or claim that may be made by its manufacturer, is not guaranteed or endorsed by the publisher.
